# Association of Sociodemographic and Perceived Environmental Factors with Public Bicycle Use among Taiwanese Urban Adults

**DOI:** 10.3390/ijerph13030340

**Published:** 2016-03-19

**Authors:** Yung Liao

**Affiliations:** Department of Health Promotion and Health Education, National Taiwan Normal University, 162, Heping East Road Section 1, Taipei 106, Taiwan; liaoyung@ntnu.edu.tw; Tel.: +886-2-7734-1722; Fax: +886-2-2363-3026

**Keywords:** public bicycle use, perceived environment, sociodemographic factors, urban adults

## Abstract

*Background*: This study examined the sociodemographic and perceived environmental factors associated with public bicycle use among Taiwanese urban adults. *Methods*: A random-digit-dialing telephone-based cross-sectional survey was administered to Taiwanese urban adults aged 20–64 years in 2015. Data on sociodemographic variables, perceived environmental factors (for attributes identified in the International Physical Activity Questionnaire-Environmental Module), and public bicycle use were obtained from 1002 adults in three cities. Adjusted logistic regression was used. *Results*: After adjustment for potential confounders, the results showed that adults aged 20–29 years (odds ratio (OR) = 4.42) with a university degree or higher (OR = 2.03) were more likely to use public bicycles. In addition, adults living in Kaohsiung City were less likely to use public bicycles (OR = 0.24). Adults who saw people being active (OR = 1.76; 95% CI: 1.05–2.86) and had positive aesthetic experiences of their environment (OR = 1.69) were more likely to use public bicycles. *Conclusions*: Our findings suggest that seeing physically active people and positive aesthetic perceptions of the environment are key factors for developing transportation policies and intervention strategies for promoting public bicycle use among Taiwanese urban adults.

## 1. Introduction

Cycling is associated with cardiorespiratory fitness and reduced risks of all-cause mortality, cardiovascular and colon cancer morbidity, and the incidence of overweight and obesity in adults [1]. In contrast to other forms of exercise and leisure-time physical activity, cycling is easier to adopt and maintain in adults’ daily routines, and allows farther travel than walking does [2,3]. Considering nearly half of all Taiwanese adults fail to achieve the minimum physical activity recommended by the World Health Organization [4,5], cycling is a potential means to promote daily physical activity among Taiwanese adults. Cycling has the additional benefits of alleviating traffic congestion, reducing air pollution and carbon emissions, and improving energy efficiency [6]. Therefore, developing effective strategies for promoting cycling is crucial.

Public bicycle share systems are recognized as a promising initiative for encouraging cycling, and are growing rapidly in the Americas, Europe, Australia, and Asia [7,8]. The use of public bicycles has been associated with higher levels of physical activity [7,9,10], as well as improvement of air quality and reduced congestion [8]. It also plays an essential role in urban transportation systems, providing short-to-medium trip lengths (1–5 km) with low trip costs compared with other travel modes [3]. Because of the multiple benefits of public bicycle use, identifying the factors affecting public bicycle use among Taiwanese adults is necessary for promoting it.

From the perspective of a behavioral epidemiological framework, a clearer understanding of the sociodemographic and perceived environmental factors associated with public bicycle use can facilitate targeting specific populations and inform urban design and planning initiatives [11]. However, despite the increasing number of public bicycle share systems, few studies have focused on the sociodemographic factors associated with public bicycle use, particularly in Asian countries. Studies from Canada and the United Kingdom have indicated that men [12,13] and young, highly educated adults [14] are more likely to use public bicycles. Moreover, although perceptions of the environment have been found to be associated with overall physical activity [15] and specific forms or contexts of physical activity such as walking, cycling or strength training [16,17,18], few studies have focused on the associations between the perceived environment and public bicycle use.

This information is critical for understanding how people perceive and understand their environment, and how perceived environmental factors could influence public bicycle use, especially regarding the perceived factors of aesthetic experiences, seeing people being active, and a sense of safety, which are difficult to measure objectively [16]. Because of the mismatches between perceived and objective environmental attributes found in previous studies [19,20], examining perceived environmental factors could inform policy makers and intervention designers about how to develop strategies that are more effective for promoting public bicycle use.

Public bicycle share systems have been implemented in three cities in Taiwan since 2009: Taipei City, New Taipei City, and Kaohsiung City. In these cities, public bicycles are intended for both tourist use and daily transit for residents. Acquiring a preliminary understanding of the associations of sociodemographic and perceived environmental factors with public bicycle use is crucial to promoting public bicycle use and, in turn, increasing physical activity and contributing toward protecting the environment in Asian countries. Therefore, this study examined the sociodemographic and perceived environmental factors associated with public bicycle use among Taiwanese urban adults.

## 2. Methods

### 2.1. Participants

In the present study, cross-sectional survey data were obtained from a random-digit-dialing telephone-based survey. The survey was conducted by a telephone research service company from September 2015 to October 2015 (mean daily temperature was 27 °C to 28 °C; monthly rainfall was 140–309 mm; and receiving on 151–208 h of sunshine per month), in three Taiwanese cities that have implemented a public bicycle share system for more than 5 years: Taipei City, the capital of Taiwan (area: 271.7 km^2^; population: 2,702,315); New Taipei City, the largest city in Taiwan (area: 2052.6 km^2^; population: 3,966,818); and Kaohsiung City, the second largest city in Taiwan (area: 2947.6 km^2^; population: 2,778,992). Currently, Taipei City, New Taipei City, and Kaohsiung City have approximately 163, 60, and 159 public bicycle stations, respectively [21,22,23]. Users of these public bicycle systems are charged by the half hour (approximately US$0.30, less than a half-hour will also be counted as a half-hour) as of 2015. In this study, a stratified and clustered multistage sampling process was used to select respondents, and the required sample size was calculated at 1067 adults with a 95% confidence level and a 3% confidence interval (CI). The interviewers had experience in administering telephone population surveys and received 2 days of training before the start of each survey. Furthermore, they administered a standardized questionnaire. A total of 5333 adults were interviewed, with 1069 completing the survey (response rate: 20.04%). Data cleaning reduced the number of participants who submitted valid data to 1068. To ensure validity, each telephone interview was no longer than 20 min to avoid the respondents feeling bored or not concentrating [24]. The telephone research service company offered no rewards for participation. Verbal informed consent was obtained before the start of the interviews, and the study protocols were reviewed and approved by the Ethics Committee of National Taiwan University (201504HM005).

### 2.2. Outcome Variable

The outcome variable was self-reported public bicycle use, identified as a valid item in a previous study [14]. The respondents indicated whether they had ever used public bicycles. If they had, the follow-up question “How many times have you used public bicycles in the previous 7 days?” was asked. Because the distribution of frequency was skewed, public bicycle use was dichotomized into indicators of use (yes) and nonuse (no) in the previous 7 days.

### 2.3. Sociodemographic Variables

Sociodemographic variables covered by the survey included gender, age, city of residence, education level, occupation type, marital status, living status, the body mass index (BMI) and bicycle ownership. Age was divided into five categories: 20–29, 30–39, 40–49, 50–59, and 60–64 years. Education level was divided into two categories: “high school degree or lower (including elementary school, junior high school, high school and vocational school degree)” and “university degree or higher”. Occupation type was divided into two categories: “not working full-time” and “working full-time”. Marital status was divided into two categories: “married” and “unmarried” (including widowed, separated, and divorced). Living status was divided into two categories: “living with others” and “living alone”. The BMI was based on self-reported weight and height, and was divided into two categories: “non-overweight” (<24 kg/m^2^) and “overweight or obese” (≥24 kg/m^2^). Bicycle ownership was dichotomized into “one or more” and “none.”

### 2.4. Perceptions of the Environment

The perceived environmental variables in the present study were measured using the Taiwanese version of the International Physical Activity Questionnaire Environmental Module (IPAQ-E). The IPAQ-E was developed as part of the International Physical Activity Prevalence Study, to investigate the relationship of perceptions of the environment with walking or cycling in neighborhoods in several countries [25,26,27]. The Taiwanese version of the IPAQ-E was used in a previous study focusing on the association between perceptions of the environment and transportation physical activity [28]. The IPAQ-E questionnaire comprises three categories of items, with seven core items, four recommended items, and six optional items [29]. Detailed descriptions of these items are available at http://www.drjamessallis.sdsu.edu/measures.html. In this study, 13 of the 17 items were utilized: (1) residential density; (2) access to shops; (3) access to public transportation; (4) presence of sidewalks; (5) presence of bike lanes; (6) access to recreational facilities; (7) safety from crime at night; (8) traffic safety; (9) seeing people being active; (10) aesthetic experiences; (11) street connectivity; (12) presence of destination; and (13) safety of cyclists in traffic. One personal item (vehicle ownership), and three optional items (sidewalk maintenance, bike lane maintenance, and safety from crime during the day) were not included in this study. As in previous studies [27,28], these 13 perceived environmental factors were converted into binary items: residential density was divided into “detached single-family housing” and “other”; the other eight items were divided into “agree” (strongly agree and somewhat agree) and “disagree” (somewhat disagree and strongly disagree).

### 2.5. Statistical Analysis

Data from 1002 Taiwanese urban adults who provided complete information for the study were analyzed. Forced-entry adjusted logistic regression for gender, age, city of residence, education level, occupation type, marital status, living status, the BMI, and bicycle ownership was conducted to examine the associations of nine sociodemographic factors and 13 perceived environmental factors associated with public bicycle use. Adjusted odds ratios (ORs) and 95% CIs were calculated for each variable. Inferential statistics was performed using IBM SPSS 22.0 software (IBM, Armonk, NY, USA), and the level of significance was set at *p* < 0.05.

## 3. Results

### 3.1. Participant Characteristics

Table 1 presents the characteristics of the respondents (mean age: 44.5 ± 12.2 years). The distribution of gender, age, and city of residence in this sample were balanced. Of the respondents, 62.9% had a university degree or higher, 69.3% had a full-time job, 66.9% were married, 95.2% were living with others, 28.1% were overweight or obese, and 72.8% owned one or more bicycles. The prevalence of public bicycle use in the previous 7 days was 10.6% in the total sample.

### 3.2. Sociodemographic Factors Associated with Public Bicycle Use in the Previous 7 Days

The ORs for using public bicycles in the previous 7 days are presented in Table 2 according to gender, age, city of residence, education level, occupation type, marital status, living status, BMI status, and bicycle ownership. Table 2 shows that urban adults aged 20–29 years (OR = 4.42; 95% CI: 1.55–12.58) with a university degree or higher (OR = 2.03; 95% CI: 1.13–3.63) were more likely to have used public bicycles in the previous 7 days. Adults living in Kaohsiung City (OR = 0.24; 95% CI: 0.12–0.47) were less likely to have used public bicycles in the previous 7 days.

### 3.3. Perceptions of the Environment Associated with Public Bicycle Use in the Previous 7 Days

Logistic regression analyses reveal that 2 of the 13 attributes of perceptions of the environment were significantly associated with public bicycle use (Table 3). Respondents who reported seeing people being active (OR = 1.73; 95% CI: 1.05–2.86) and had positive aesthetic experiences (OR = 1.84; 95% CI: 1.16–2.92) were more likely to have used public bicycles in the previous 7 days.

## 4. Discussion

Based on our research, this is the first study from an Asian country to examine the associations of sociodemographic and perceived environmental factors with public bicycle use among urban adults. The findings have crucial implications for identifying potential environmental and policy interventions, suggesting that targeting adults who are older and have lower levels of education, especially those living in Kaohsiung City, as well as optimizing neighborhood aesthetics and increasing the visibility of people being active may be effective for promoting public bicycle use in urban adult populations. These results may have crucial implications for policy makers or intervention designers developing intervention strategies for promoting public bicycles as a main means of daily transit

The present study revealed two aspects of perceived environmental attributes associated with higher levels of public bicycle use: seeing people being active and positive aesthetic experiences of the environment. Our results support previous findings that the associations between positive aesthetic experiences of the environment and specific forms of physical activity are positive in various countries [16,17,28,30]. In addition, a positive relationship was revealed between perceived environmental factors and public bicycle use. A possible explanation for this result is that aesthetic factors—such as attractively landscaped, well-maintained, and clean environments [31]—are strong incentives because they attract people and motivate them to use public bicycles more for short-to-medium distance trips [3]. This finding is crucial for urban design because optimizing aesthetic perceptions could be less expensive than other structural manipulations of the built environment [17,32,33]. Moreover, seeing people being active, a sociocultural environment factor, was found to be associated consistently with moderate-to-vigorous physical activity in adults [34], and is also positively associated with public bicycle use. This could be explained by a key concept from social cognitive theory: observational learning [35]. This suggests that public bicycle use could be motivated by others being active, such as other users of public bicycles and other forms of active transportation. Further research using both qualitative and quantitative method is required to elucidate these associations and explanations. Thus, the implications of these findings are that making neighborhoods more pleasant and enhancing the visibility of people being active through urban design and policy initiatives may attract and motivate urban residents to use public bicycles.

Another finding of this study is that three sociodemographic characteristics, namely age, education level, and city of residence, were associated with public bicycle use. Consistent with the findings of a Canadian study [14], younger and more educated Taiwanese urban adults were more likely to have used public bicycles. This is because youth and education are recognized as consistent demographic correlates of physical activity in adults [36]. The present findings may extend relevant literature, in that young and highly educated adults are more likely to use public bicycles. Furthermore, our findings indicate that adults living in Kaohsiung City were less likely to use public bicycles. A possible reason for this result could be that the prevalence of using private vehicle as a main means of transportation was high (estimated at 85.3%, mainly because of the convenience) in adults of Kaohsiung City, compared with Taipei City (47.4%) and New Taipei City (61.2%) [36]. Although public transport system and cycling infrastructure of Kaohsiung City has been implemented in recent years, only 14.7% of Kaohsiung City residents reported using active modes of transportation, and 9.1% used public transportation as their main means of travel [37]. Thus, future intervention studies should develop strategies for promoting public bicycle use among adults of Kaohsiung City. These findings will inform policy makers and intervention designers that efforts to promote the use of public bicycle share systems should be focused on adults who are older, have lower levels of education, and live in Kaohsiung City.

Several limitations of the current study should be considered. First, because a cross-sectional design was used, causality between perceived environmental factors and public bicycle use could not be determined. Second, the main measurements were self-reported and could be subject to bias [38]. Third, potential confounders such as self-selection of neighborhoods and seasonal factors (*i.e.*, weather condition) were not examined in this study. Finally, this study had a limited representative sample because it relied on a telephone-based survey; therefore, sections of the population without a household telephone (approximately 5.3% in 2013) were impossible to reach [39]. Moreover, compared with national data [40,41,42], the respondents in the present study were more highly educated and had a higher prevalence of being overweight. Thus, the results of the present study may be less applicable to the general population.

## 5. Conclusions

Consistent with studies conducted in Western countries, our study demonstrates that younger adults with higher levels of education are more likely to use public bicycles. Our findings suggest that increasing both the visibility of people being active and positive aesthetic experiences are crucial for developing transportation policies and intervention strategies for promoting public bicycle use among Taiwanese urban adults.

## Figures and Tables

**Table 1:** Basic characteristics of respondents (*n* = 1002).

Basic Characteristics	Total	Public Bicycle Use		*p*-Value
		Yes	No	
N (%)	1002	106 (10.6%)	896 (89.4%)	
Gender				0.25
Men	496 (49.5%)	54.7%	48.9%	
Women	506 (50.5%)	45.3%	51.1%	
Age (year)				<0.001 **
20–29	138 (13.8%)	32.1%	11.6%	
30–39	226 (22.6%)	24.5%	22.3%	
40–49	249 (24.9%)	21.7%	25.2%	
50–59	257 (25.6%)	15.1%	26.9%	
60–64	132 (13.2%)	6.6%	14.0%	
Residential city				<0.001 **
Taipei city	334 (33.3%)	48.1%	31.6%	
New Taipei city	341 (34.0%)	39.6%	33.4%	
Kaoshiung city	327 (32.6%)	12.3%	35.0%	
Educational level				<0.001 **
High school degree and lower	372 (37.1%)	17.0%	39.5%	
University and higher	630 (62.9%)	83.0%	60.5%	
Occupational type				0.14
Not full-time	308 (30.7%)	24.5%	31.5%	
Full-time	694 (69.3%)	75.5%	68.5%	
Marital status				0.001 *
Not married	332 (33.1%)	48.1%	31.4%	
Married	670 (66.9%)	51.9%	68.6%	
Living status				0.65
Living alone	48 (4.8%)	5.7%	4.7%	
Not living alone	954 (95.2%)	94.3%	95.3%	
Body Mass Index				0.04 *
Non-overweight	720 (71.9%)	80.2%	70.9%	
Overweight/obese	282 (28.1%)	19.8%	29.1%	
Bicycle ownership				0.83
One or more	273 (27.2%)	26.4%	27.3%	
None	729 (72.8%)	73.6%	72.7%	

* *p* < 0.05; ** *p* < 0.001.

**Table 2:** Sociodemographic factors associated with public bicycle use among Taiwanese adults.

Sociodemographic Factors	Total	*p*-Value
Gender		
Men	1.00	
Women	0.88 (0.56–1.36)	0.57
Age (year)		
60–64	1.00	
50–59	1.07 (0.42–2.75)	0.88
40–49	1.39 (0.54–3.58)	0.49
30–39	1.50 (0.56–3.95)	0.41
20–29	4.42 (1.55–12.58)	0.005 *
Residential city		
Taipei city	1.00	
New Taipei city	1.00 (0.63–1.59)	0.99
Kaoshiung city	0.24 (0.12–0.47)	<0.001 **
Educational level		
High school degree and lower	1.00	
University and higher	2.03 (1.13–3.63)	0.01 *
Occupational type		
Not full-time	1.00	
Full-time	1.32 (0.78–2.25)	0.29
Marital status		
Not married	1.00	
Married	1.12 (0.62–2.05)	0.69
Living status		
Living alone	1.00	
Not living alone	0.98 (0.37–2.57)	0.97
Body Mass Index (kg/m^2^)		
Non-overweight	1.00	
Overweight/obese	0.67 (0.39–1.15)	0.14
Bicycle ownership		
One or more	1.00	
None	1.30 (0.80–2.12)	0.27

* *p* < 0.05; ** *p* < 0.001.

**Table 3:** Perceived environmental factors associated with public bicycle use among Taiwanese adults.

Perceived Environmental Factors			Public Bicycle Use	*p*-Value
	N	%	OR (95% CI)	
Residential density				
High	954	95.2%	0.54 (0.19–1.51)	0.24
Low	48	4.8%	1.00	
Access to shops				
Good	898	89.6%	1.02 (0.49–2.12)	0.95
Poor	104	10.4%	1.00	
Access to public transport				
Good	933	93.1%	1.98 (0.56–6.62)	0.29
Poor	69	6.9%	1.00	
Presence of sidewalks				
Yes	767	76.5%	1.33 (0.75–2.34)	0.31
No	235	23.5%	1.00	
Presence of bike lanes				
Yes	466	46.5%	1.46 (0.94–2.26)	0.09
No	536	53.5%	1.00	
Access to recreational facilities				
Good	894	89.2%	0.85 (0.41–1.77)	0.67
Poor	108	10.8%	1.00	
Crime safety at night				
Not safe	206	20.6%	0.91 (0.52–1.57)	0.74
Safe	796	79.4%	1.00	
Traffic safety				
Not safe	383	38.2%	1.12 (0.73–1.74)	0.58
Safe	619	61.8%	1.00	
Seeing people being active				
Yes	689	68.8%	1.73 (1.05–2.86)	0.03 *
No	313	31.2%	1.00	
Aesthetics				
Yes	600	59.9%	1.84 (1.16–2.92)	0.009 *
No	402	40.1%	1.00	
Connectivity of streets				
Yes	771	76.9%	1.44 (0.81–2.57)	0.21
No	231	23.1%	1.00	
Presence of destination				
Yes	829	82.7%	1.50 (0.79–2.84)	0.21
No	173	17.3%	1.00	
Traffic safety of byclists				
Not safe	527	52.6%	0.71(0.46–1.09)	0.12
Safe	475	47.4%	1.00	

* *p* < 0.05.
